# ZnO Nanoparticles: Advancing Agricultural Sustainability

**DOI:** 10.3390/plants14152430

**Published:** 2025-08-05

**Authors:** Lekkala Venkata Ravishankar, Nidhi Puranik, VijayaDurga V. V. Lekkala, Dakshayani Lomada, Madhava C. Reddy, Amit Kumar Maurya

**Affiliations:** 1 Production and Plantations, Red Otter Farms Pvt Ltd., Nainital 263159, Uttarakhand, India; 2 Department of Life Sciences, Yeungnam University, Gyeongsan 38541, Republic of Korea; 3 Department of Genetics and Genomics, Yogi Vemana University, Kadapa 516005, Andhra Pradesh, India; lvijayadurga123@gmail.com (V.V.V.L.); dlomadayvu@gmail.com (D.L.); 4 Department of Biotechnology and Bioinformatics, Yogi Vemana University, Kadapa 516005, Andhra Pradesh, India; cmadhavareddy@gmail.com; 5 Department of Plant Pathology, Teerthanker Mahaveer University, Moradabad 244001, Uttar Pradesh, India; mauryaamit856@gmail.com

**Keywords:** nanotechnology, sustainable agriculture, nanonutrients, nanobiofungicides

## Abstract

Micronutrients play a prominent role in plant growth and development, and their bioavailability is a growing global concern. Zinc is one of the most important micronutrients in the plant life cycle, acting as a metallic cofactor for numerous biochemical reactions within plant cells. Zinc deficiency in plants leads to various physiological abnormalities, ultimately affecting nutritional quality and posing challenges to food security. Biofortification methods have been adopted by agronomists to increase Zn concentrations in crops through optimal foliar and soil applications. Changing climatic conditions and conventional agricultural practices alter edaphic factors, reducing zinc bioavailability in soils due to abrupt weather changes. Precision agriculture emphasizes need-based and site-specific technologies to address these nutritional deficiencies. Nanoscience, a multidimensional approach, reduces particle size to the nanometer (nm) scale to enhance their efficiency in precise amounts. Nanoscale forms of Zn^+2^ and their broad applications across crops are gaining attention in agriculture under varied application methods. This review focuses on the significance of Zn oxide (ZnO) nanoparticles (ZnONPs) and their extensive application in crop production. We also discuss optimum dosage levels, ZnONPs synthesis, application methods, toxicity, and promising future strategies in this field.

## 1. Introduction

Mineral nutrition is an intriguing aspect of crop physiology in agriculture, essential for enhancing crop productivity [1]. The changing climate trends raise concerns about the nutritional and quality parameters of food due to plant susceptibility to various abiotic and biotic stresses [2]. The green revolution emphasized conventional farming practices that boosted food production but overlooked potential environmental hazards [3,4]. The breeding of modern cultivars with yield-enhancing traits, combined with the widespread use of plant protection chemicals and heavy application of inorganic fertilizers, has progressively degraded soil fertility and nutrient status [5]. Soil serves as a vital component of agricultural food systems [6], as the bioavailability of diverse structural and functional elements creates a healthy medium necessary for seed germination, plant growth, and fruitful yields [7]. Soil degradation negatively impacts fertility and raises concerns about the future of food security [8,9]. Changes in soil pH levels mainly affect nutrient availability due to altered ionic concentrations in the root zone, which hampers nutrient uptake and may worsen malnutrition and human health issues. Globally, 854 million people suffer from deficiencies in protein and micronutrients (e.g., Zn, Fe, Se, B, I), with hidden hunger especially prevalent among small children in a population of 3.7 billion [10]. The “Criteria of Essentiality” classifies plant nutrients into macro- and micro-elements. While macronutrients are essential structural components that enhance crop productivity and are applied in bulk, micronutrients receive comparatively little attention in crop production systems [11,12]. Excessive use of primary elements, compounded by climate challenges, exposes plants to several abiotic and biotic stresses, ultimately reducing yields due to poor performance [13]. To counteract and overcome environmental stressors, plants have developed various resistance mechanisms, including both adaptive and innate responses, which alter the internal micronutrient pool and may make them more susceptible to pathological conditions [14,15]. Micronutrients play a critical role in plant health, from the external rhizosphere surrounding the roots to the internal ultrastructural units of the plant [16], and deficiencies in these nutrients can significantly affect plant stability and growth [17].

## 2. Micronutrients: Plant Growth and Development

Nutritional insecurity is an alarming global issue, with food quality compromised by low micronutrient levels—raising serious concerns about malnutrition and undernutrition-related health problems [18,19]. The agri-food chain plays a vital role in delivering essential nutrients to both plants and humans [20]. In light of these challenges, an integrated approach is needed to sustainably improve the nutritional quality of food and help reduce hidden hunger [21,22]. Given the increasing demand for higher crop yields, the application of micronutrients has gained significant importance [23]. Micronutrients—such as boron (B), copper (Cu), chlorine (Cl), iron (Fe), zinc (Zn), manganese (Mn), and molybdenum (Mo)—are essential for plant development, even though they are absorbed in relatively small quantities [24]. These elements are fundamental to plant metabolism and growth, and their deficiency can lead to plant diseases, ultimately decreasing both the quality and quantity of food produced [17]. The present review emphasizes the importance of Zn in plant growth and development across various applications. Notably, structural modifications of zinc at the nanoscale have shown a greater impact on the fertilizer use efficiency (FUE) of other nutrients due to their synergistic effects. This review also highlights Zn’s role in defense signaling mechanisms against a range of abiotic and biotic stresses, underlining the significance of nanostructured zinc elements for enhancing food system efficiency through a sustainable approach has been described in Table 1.

### 2.1. Zinc as Biocatalyst

Enzymes act as biocatalysts for numerous biochemical reactions in plant cells. Among the six major enzyme classes—oxidoreductases, transferases, hydrolases, lyases, isomerases, and ligases—zinc is the only metal required for the proper function of all [38]. As a transition element, zinc remains in the Zn^+2^ form and is not subject to valence changes; it primarily functions as a divalent ion in metalloenzymes, serving as a linkage between enzymes and their corresponding substrates. Zn forms tetrahedral complexes with N and O and is particularly linked to S in various organic compounds [39]. Zn deficiency disrupts several plant metabolic processes, including carbohydrate and protein synthesis, and negatively affects membrane integrity. Zn-dependent enzymes involved in carbohydrate metabolism in foliage are especially impacted [40]. Furthermore, Zn deficiency significantly reduces the activity of carbonic anhydrase enzymes [41]. Zinc acts as a metallic cofactor for over 300 enzymes in biological systems and binds to cytoplasmic proteins essential for cell signaling and communication, often attaching to DNA. The structural diversity of zinc, especially its role in forming zinc finger motifs, highlights its critical and versatile function in biological systems [42].

### 2.2. Zinc in Hormone Regulation

Tryptophan, a precursor of the auxin growth hormone [43], is involved in the biosynthesis of indoleacetic acid (IAA) [44], which represents a major pathway for auxin production in plants [44,45]. Auxin biosynthesis involves a two-step pathway, with the YUCCA (YUC) family of flavin monooxygenase enzymes acting as key components. Overexpression of YUC in Arabidopsis leads to auxin overproduction. Genetic and physiological studies have demonstrated that YUC genes catalyze a rate-limiting step in the Trp-dependent auxin biosynthesis pathway, confirming that both YUCs and TRYPTOPHAN AMINOTRANSFERASE OF ARABIDOPSISs (TAA) are essential for auxin synthesis and plant development [46]. YUC-mediated auxin biosynthesis is dependent on the activity of TAA enzymes [47]. The initial step involves the removal of an amino group from Trp by TAA, which generates indole-3-pyruvate (IPA). This intermediate then undergoes oxidative decarboxylation by the YUC family of flavin monooxygenases- a two-step pathway central to plant developmental processes [48]. Zinc plays a crucial role by activating tryptophan synthetase, which is responsible for producing tryptophan used in IAA biosynthesis, particularly in meristematic tissues involved in phototropic growth. Zn deficiency impairs the production and expression of genes related to IAA synthesis due to inactivation of tryptophan synthetase, an enzyme whose activation depends on metallic Zn [49]. This enzyme catalyzes the union of serine and the indole ring, a critical step in IAA synthesis. It is referred to as part of the “three-ring circus,” a term describing the three key proteins involved in IAA production [50]. Visual symptoms of Zn deficiency may be attributed to reduced IAA levels or inhibited IAA synthesis.

### 2.3. Zinc for Abiotic and Biotic Stress

#### 2.3.1. Abiotic Stress Regulation

Zn interacts with various plant hormones to mitigate abiotic stress by elevating the expression of stress-responsive proteins, maintaining cell membrane integrity with the support of antioxidants, and countering the effects of drought [51]. Seed priming with Zn has been shown to enhance germination percentage and yield in maize, wheat, and chickpea under different environmental conditions [52,53]. During drought, plumule length and seedling weight are adversely affected due to inadequate nutrient supply to the embryo. However, seed priming with Zn improves the expression of IAA and GA3 under drought stress, promoting plumule elongation and increased seedling weight [53]. Maintaining the structural integrity of the cell membrane is a core strategic mechanism in plant drought tolerance during stress conditions [54,55]. Adequate Zn supply stabilizes membranes by counteracting reactive oxygen species (ROS), resulting in improved leaf area, chlorophyll content, and photosynthetic pigment levels, thereby enhancing yields [56,57,58]. Antioxidant enzymes such as superoxide dismutase (SOD) and peroxidase (POD) play a key role in reducing oxidative stress by lowering malondialdehyde (MDA) content and minimizing ion leakage [59]. Osmotic adjustment is another critical strategy, contributing to higher turgor pressure and improved water retention under drought conditions [60]. The accumulation of low-molecular-weight compounds in plant cells, such as proline, glycine betaine, soluble sugars (SS), and soluble proteins, acts as osmolytes that support the normal functioning of cell organelles, thereby maintaining assimilation efficiency during stress [61]. Zn finger proteins constitute one of the largest families of transcription factors. Due to their finger-like structure enabling DNA binding, they have been categorized into nine subfamilies based on their conserved cysteine (Cys) and histidine (His) motifs: Cys2/His2-type (C2H2), C3H, C3HC4, C2HC5, C4HC3, C2HC, C4, C6, and C8. Among these, the C2H2-type is the most extensively studied, known for its role in plant growth, development, and signal transduction under various abiotic stresses [62,63]. The Zat12 zinc finger protein is associated with resistance to several biotic and abiotic stresses and is believed to mediate oxidative stress signaling in Arabidopsis (*Arabidopsis thaliana*) [64]. External or foliar application of Zn during drought conditions has been shown to enhance the expression of Zn finger proteins, which in turn stimulates the production of SS and proline—both key components in plant drought tolerance mechanisms [65,66,67].

K+ ions are crucial for stomatal conductance, regulating transpiration losses during drought. Zn application can improve K+ influx efficiency in higher plants, thus aiding in the regulation of transpiration rates [68]. As a catalytic element, Zn deficiency negatively affects carbonic anhydrase activity, thereby impairing the plant’s assimilation capacity. Zn also plays a role in repairing Photosystem II (PSII), which supports the structure of the abundantly present RUBISCO enzyme, ultimately enhancing photosynthesis under drought conditions [69,70]. Moreover, Zn application can reduce the accumulation of substomatal CO_2_, which otherwise limits the photosynthetic rate [71].

#### 2.3.2. Biotic Stress

Zn plays an important role in defense signaling in response to biotic stress. This metallic enzyme elevates defensive processes against pathogens [72]. The defensive role of Zn proteins increases tolerance levels or helps avoid the effects of invading pathogens, though it is not effective in every crop, as it can create susceptibility in some. Disease studies have revealed the importance of Zn nutrition in plant defense responses under different applications of Zn-based compounds and nanoparticles (NPs), which are effective in managing pathogen growth. These serve as alternatives to conventional agro-pesticides, offering greater efficacy at lower doses [73]. Zn applications also mitigate the risks posed by soilborne pathogens such as *Fusarium solani*, *Rhizoctonia solani*, and *Macrophomina phaseolina* in tomato; root damping in wheat; and dry root rot in chickpea, cowpea, and Medicago sativa [74]. Zn deficiency in soil and leaves increases susceptibility to Phytophthora pod rot (or black pod) in cocoa, particularly in Papua New Guinea [75]. Meta-analysis of various R gene proteins with Zn finger domains across crop species revealed that, out of seventy R genes, twenty-six contained Zn finger domains along with NBS-LRR. It was also found that Zn finger domain lengths within R genes varied from 11 to 84 amino acids [76]. In transgenic cotton, the cloned DNA from *Gossypium hirsutum* zinc finger protein 1 (GhZFP1) encodes a novel CCCH-type zinc finger protein isolated from salt-induced *G. hirsutum*. Overexpression of GhZFP1 in transgenic tobacco enhanced salt tolerance and resistance to Rhizoctonia solani, which causes damping-off in cotton [77]. The OsLOL2 gene in transgenic rice, orthologous to the LSD1 gene in *Arabidopsis thaliana*, encodes a 163-amino acid polypeptide with LSD1-like zinc finger domains sharing 74.5% identity with LSD1, conferring resistance to rice bacterial leaf blight [78]. Zinc domain proteins of R genes in rice, such as Pi54, confer durable resistance to rice blast disease caused by *Magnaporthe oryzae* through upregulation of guard genes and activation of transcription factor genes [79]. Stripe rust tolerance in wheat is mediated by SA-dependent responses and hypersensitive reaction responses involving zinc-binding proteins [80].

## 3. Nanotechnology

**Figure 1:**
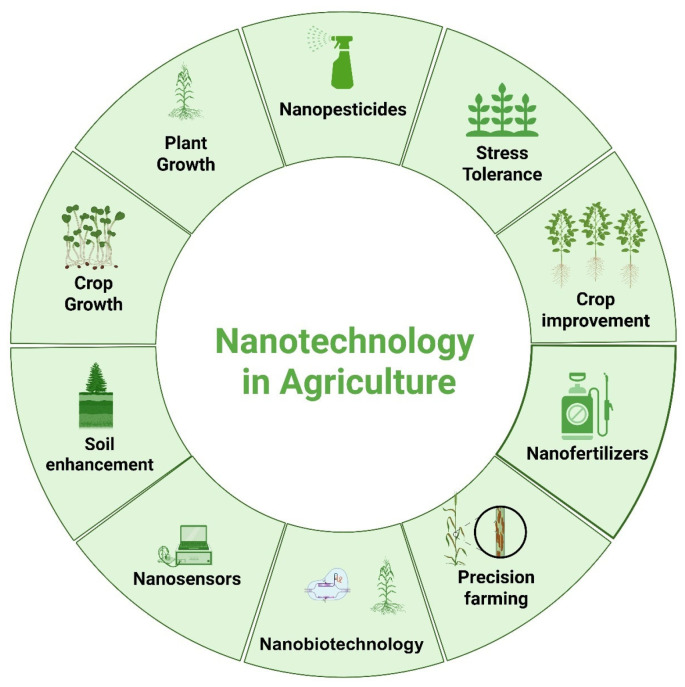
The role of nanotechnology is clearly explained through its multiple applications in the agriculture sector via alteration in particle structures; this represents the core concept of precision science, where all productive and protective measures are followed for efficient nutrient delivery while safeguarding the environment.

Nanotechnology is recognized as one of the rapidly advancing technologies of the 21st century, integrating chemistry, physics, and biology. It involves the production, design, and characterization of materials by controlling size and shape at the nanometer scale (1–100 nm) [81]. Major applications of nanomaterials, including atom manipulation at the nanoscale, span various interdisciplinary fields such as drug delivery, diagnostics, and tissue engineering. Due to their small size, nanoparticles can target ligand sites, specifically at inflammation sites. Nanoformulated drugs offer several advantages over conventional medicines, including increased bioavailability and reduced side effects [82]. Metal nanoparticles have gained considerable attention, and their synthesis can be achieved through biological, chemical, and physical processes. These are generally categorized into top-down and bottom-up approaches. The top-down approach involves breaking down bulk materials into nanoscale forms, with examples including sputtering, ball milling, thermal decomposition, and laser ablation. In contrast, the bottom-up approach is constructive, forming nanoparticles from simpler molecules. Examples include chemical vapor deposition, spinning, sol–gel methods, and biological synthesis [83]. Applications of nanotechnology in various agricultural sectors are illustrated in Figure 1. The ecofriendly synthesis of metal or metal oxide nanoparticles using plant extracts (such as stems, flowers, seeds, leaves, and bark) presents a novel, green alternative to conventional synthesis methods that involve toxic chemicals [84]. One of the key advantages of using eco-synthesis over other methods is that natural metabolites act as reducing agents, converting bulk metal salts into metal ions. Major phytochemicals involved in this process include terpenoids, ketones, phenols, amides, and aldehydes [85]. Nanoparticles have been synthesized using various plant extracts, as listed in Table 2.

## 4. Mode of Applications of Zn Oxide (ZnO) Nanoparticles in Crop Protection

### 4.1. Seed Priming

Seed priming is a modest yet effective method to enhance seed germination by activating hydrolytic enzymes responsible for this process. It involves submerging seeds in aqueous solutions containing nutrients, hormones, and enzymes, which support proper and rapid seed growth [96,97]. The mechanism of seed priming includes several steps that trigger enzyme activity. Germination in non-dormant seeds proceeds through three distinct phases: (I) imbibition, (II) the lag or activation phase, and (III) radicle protrusion or rehydration [98].

### 4.2. Foliar Sprays

**Figure 2:**
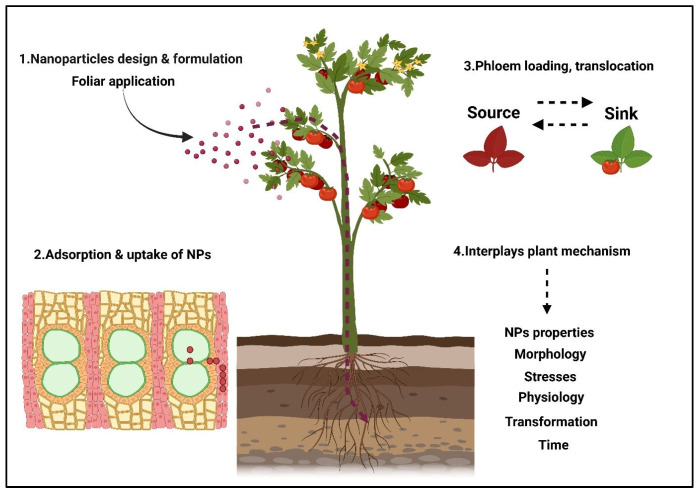
(1) Nanostructured ZnO nanoparticle (ZnONP) application and its mode of entry through site-specific application. (2) Applied nanoparticles penetrate the leaf surface at different locations via symplastic or apoplastic pathways. (3) Symplastic or apoplastic movement directs and translocates either from source to sink or vice versa. (4) Finally, they target and counteract different stresses through physiological interactions, transforming over time.

Foliar applications are considered highly important in agriculture. However, it is estimated that up to 50% [99] of foliar-applied nutrients and over 95% [100] of pesticides under agroecosystem conditions do not reach their intended targets, leading to waste. This inefficiency contributes to soil pollution, antibiotic resistance, and runoff into natural water bodies [101]. Nanoformulated sprays have been applied externally, allowing nutrients to enter leaves directly through stomata and into the cytosol. This method is especially effective for nutrients that are immobile or unavailable under varying soil pH conditions, which can cause nutrient starvation despite elemental abundance [102]. The adhesion ability of NPs is crucial and highly dependent on the functional groups present on the NPs [103,104]. Specific chemical compounds on the leaf surface, such as glucosides, proteins, and wax deposits, also affect this adhesion [105,106]. Smaller NPs, due to their precision and higher activity, are generally more effective than larger NPs, which have a greater surface area but reduced reactivity. Limitations related to NP size affect their uptake and distribution in plants due to issues of stability and aggregation. NPs within the size range of 50–60 nm have been successfully absorbed by plants through foliar application [107]. Large structures, such as lipid-based nano-fertilizers, have also been detected inside plant tissues following foliar deposition, with lipid NPs ranging from 150 to 300 nm [108] and liposomal formulations around 100 nm in size [109]. The entry of NPs into plant cells has been addressed through various pathways, including cuticular entry [110], symplastic transport [111], and apoplastic movement [112], enabling distribution from foliar surfaces to unexposed plant parts, as illustrated in Figure 2.

## 5. Applications of Zinc Nanoparticles on Various Growth Parameters

### 5.1. Plant Growth and Development

The external application of ZnONPs has been shown to alter phytohormone levels in a site-specific manner upon exposure [113]. Previous studies have reported variations in the concentrations of cytokinins (CKs), auxins, ABA, SA, and IA across different plant regions—including apical, basal, and lateral parts. Specifically, CKs and auxins were downregulated, while salt tolerance increased due to the deposition of cis-zeatin in the root zone [113]. Phenotypic changes in plants, such as variations in root and shoot length, were observed under dose-dependent treatments with ZnONPs [113]. ZnONPs applications at low concentrations—such as 1.5 ppm seedling development, resulting in have demonstrated promising effects on chickpea higher biomass and reduced malondialdehyde content [114]. Field-level experiments have further revealed that multiple applications of ZnONPs at low concentrations can yield significantly better results compared to standard ZnSO_4_ treatments, with pod yields increasing by more than 29.5% [115].

Foliar application of nano-chelated zinc at 200 and 100 ppm significantly improved various agronomic and quality traits in grapes. The 200-ppm treatment was superior in enhancing leaf count, leaf area, chlorophyll content (a, b, total), total carbohydrates in branches, leaf dry weight, IAA concentration, cluster number, cluster width, berry number, 100-berry size, total yield, TSS, TSS/TA ratio, total sugars (glucose, fructose), β-carotene, anthocyanins, phenols, and juice pH. Meanwhile, the 100-ppm treatment excelled in cluster weight, 100-berry weight, rachis weight, berry set percentage, juice percentage, and the content of tartaric and malic acids, as well as vitamin C. In terms of nutrient uptake, 200 ppm significantly increased phosphorus, potassium, and zinc concentrations in leaf petioles, while 100 ppm was more effective for nitrogen uptake. Overall, both concentrations positively influenced grape quality and productivity, 200 ppm being more effective across most parameters, while 100 ppm offered specific advantages in yield and biochemical traits [116].

Soil application of 10 mg/kg and foliar spraying with 10 ppm ZnONPs, applied three times, showed the best performance in mulberry, promoting leaf emergence, survival rate, and sprouting percentage. The 10 mg/kg soil application of ZnONPs contributed to ROS detoxification and maintained cell membrane integrity through enhanced antioxidant enzyme activity. Zinc content in leaves increased significantly—by 147.50%, 179.49%, and 171.99%—in different parts of treated cuttings [117]. ZnO and ZnONPs demonstrate a wide range of functional activities across various industrial applications [118]. Studies have emphasized the importance of ZnONPs in seed decontamination [119], biomass accumulation, nutritional enhancement, and zinc bioaccumulation, while also stimulating secondary metabolism and improving yield [119,120,121]. At optimal dosages, they confer tolerance to various abiotic stresses, including salinity in sorghum [122] and cadmium toxicity in wheat [123], tomato, and maize [124,125]. Bulk ZnO (B-ZnO) and ZnONPs were evaluated, with results indicating more promising outcomes for ZnONPs in promoting accelerated growth during the reproductive phase, showing an average increase of 15% over the control. These nanostructured elements enhanced crop yield in terms of fruit number and fresh mass [126]. The synthesis and application of ZnONPs from different zinc sources showed differential responses at three concentration levels—0, 250, and 500 mg kg^−1^ using nano-Zn chelate, Zn sulfate, and nano-ZnO. Among these, Zn chelate demonstrated a significant effect on total phenol and soluble sugar content in grapes at 500 mg/kg^−1^. Biochemical parameters and potassium content also increased markedly in response to nano-Zn chelate application [127]. Seed priming with ZnONPs at varying concentrations (0, 5, 10, 15, 25, and 50 ppm) produced favorable results, with improvements observed in agronomic traits such as plant height, chlorophyll content, and 1000-seed weight across concentrations when compared to the control. Notably, ZnONPs at 25 ppm proved most effective under water-deficit conditions [128].

### 5.2. Nano Zinc Role in Abiotic Stress

**Figure 3:**
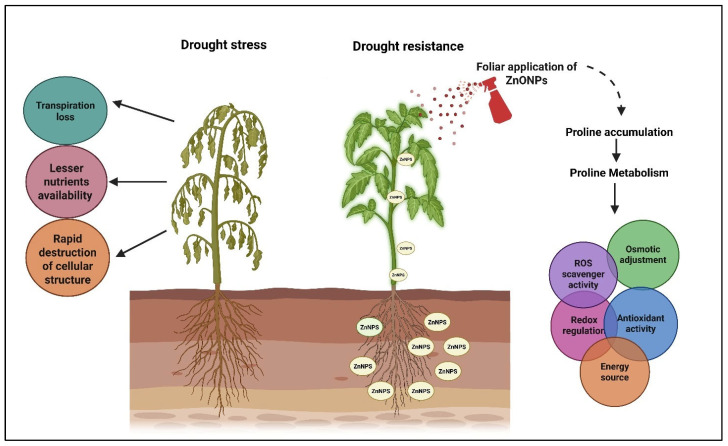
Importance of different ZnO nanoparticles (ZnONPs) in combating various abiotic stresses and their physiological responses, observed through changes such as drought-resistant protein proline expression, along with cellular responses like reactive oxygen species (ROS) scavenging, osmotic adjustment to prevent water loss, increased antioxidant activity, and conservation of metabolic energy sources.

Emphasis on and sound scientific background of ZnONPs, supported by nearly twenty years of research and approximately 6932 scientific records available on the Web of Science website, highlight the green synthesis of ZnONPs as extensively studied in countries like China, India, and Iran. This emerging technology is being developed to address present-day challenges in agriculture through a sustainable approach [129]. Foliar application of ZnO NPs at 100 ppm under heat stress conditions in mango crops enhanced yield and nutritional parameters compared to the conventional application of zinc chelate and zinc sulfate, applied during the first week of January and repeated at four-week intervals [130]. ZnONPs at 300, 600, and 1000 mg/kg soil influenced enzymatic and non-enzymatic antioxidant parameters in tomato [131]. Foliar application of ZnO and BO nanoparticles at 250, 500, and 1000 ppm showed significant effects on foliage number, leaf area, shoot length, and root length at three regular intervals, with a notably lower percentage of fruit cracking observed when applied at 1000 ppm [132]. Greenhouse studies under different saline stress conditions in pot culture, using three foliar application levels of nano-zinc at 100 and 200 ppm and a control under 10%, 20%, and control saline conditions, revealed that foliar spray of ZnONPs at 200 ppm helped cotton plants **withstand** saline stress, suggesting potential use of saline water in cotton cultivation [133]. Nanoformulation of ZnSO_4_ enhances plant growth and helps overcome salinity stress in rice; ZnSO_4_NPs applied at 10 mg/kg of soil reduced chemical attributes such as sodium and sodium adsorption ratio [134]. Saline stress in *Phaseolus vulgaris* underscores the importance of nano-ZnO particles at 50, 100, 200, and 250 ppm. Nano-priming of seeds enhances calcium uptake in terminal leaf parts and reduces Na accumulation in aerial tissues, promoting robust growth. This demonstrates the role of ZnONPs as a sustainable, climate-resilient solution for *P. vulgaris* L. under elevated saline conditions. Biofortification and FUE were elucidated in wheat to evaluate the impact of metallic nanoparticles with bioagents—Zinc-solubilizing bacteria *Pseudomonas aeruginosa* (YZn1) and *Stenotrophomonas maltophilia* (WZn1)—used in combination with ZnO nanoparticles. Soil application of ZnSB combined with a foliar spray of ZnONPs significantly (*p*  ≤  0.05) improved yield and yield traits [135]. Seed priming with 1000 ppm ZnONPs in groundnut enhanced both seed germination and seedling vigor, also promoting early flowering and higher chlorophyll content [115]. TPP-Chitosan and TPP-Chitosan-Zinc Oxide nanoparticles were shown to modulate phosphorus leaching losses through Tripolyphosphate. Additionally, TPP-Chitosan-ZnO increased wheat grain yield by 21% and 30%, with higher Zn concentration observed in the TPP-Chitosan nanoparticle treatment [136]. Mycosynthesized ZnO nanoparticles improved plant phenological parameters; as a cofactor in P-solubilizing enzymes, nano-ZnO increased phosphate and phytase activity by 84–108% [137]. Figure 3 illustrates the effect of ZnO nanoparticles under drought conditions and Table 3 represents Zinc nanoparticle applications and their induced changes in different crops.

### 5.3. Applications of Nano-Zinc in Biotic Stress

#### 5.3.1. Fungus

The effect of ZnONPs on black scurf of potato caused by *Rhizoctonia solani* shows a maximum inhibition rate of mycelium at 3% under in vitro conditions, with the lowest recorded disease severity at 4.73% compared to the control [144]. The antifungal activity against fungal wilt in tomato at three concentrations—3000, 1500, and 100 ppm—resulted in plant heights ranging from 166 to 175 cm under greenhouse trials, with a disease scale of 0.4–0.8 and an incidence rate between 20% and 40% [145]. Mycosynthesized ZnONPs exhibited strong antifungal activity against *Fusarium oxysporum*, reducing disease severity by 75% and enhancing recovery by improving morphological and metabolic indicators [146]. Ecofriendly management of *Fusarium* wilt using hydrogel-based ZnO and watermelon peel waste (particle size: 10–20 nm) demonstrated promising antifungal activity under in vitro conditions [147]. Brown spot, a major airborne pathogen in rice, was controlled through foliar application of ZnONPs at 10 and 25 ppm, reducing infection, sporulation, and fungal colony formation, with notable suppression at 25 and 50 ppm concentrations under in vitro conditions [148]. Green synthesis of ZnO nanoparticles (20–30 nm) using *Terminalia bellerica* (Baheda) showed both antifungal and antibacterial activity against *Alternaria* blight/leaf spot disease in *Brassica* species [149].

#### 5.3.2. Bacterial

Defense response biochemical constituents such as SOD, catalase (CAT), ascorbate peroxidase (APX), phenylalanine ammonia-lyase, glutathione (GSH), proline, H_2_O_2_, and malondialdehyde (MDA) levels in beetroot increased under ZnO and TiO_2_ application against *P. betavasculorum*, *X. campestris* pv. *beticola*, and *P. syringae* pv. *aptata* [150]. ZnO nanoparticles showed highly significant results under foliar application @ 10 mL of 0.1 mg mL^−1^ in lentil, whereas the combination of ZnO with Rhizobium showed non-significant results and negatively affected nodulation [151]. High-potential antibacterial activity of spherical-shaped ZnO and ZnO_2_ NPs with a shell diameter of 5 nm effectively reduced disease symptoms by 7–19% for medium and large HLB (huanglongbing, or citrus greening) under field conditions. In vitro studies also indicated high antibacterial efficiency at 9–18 μg mL^−1^, with biofilm inhibition at 50 μg mL^−1^ [152]. *Xylella fastidiosa*, a xylem-inhibiting bacterium causing severe economic losses in many commercial and horticultural crops, showed a 100% reduction under 60 ppm of ZnONPs in Pierce’s disease medium. Greenhouse studies of *X. fastidiosa* subsp. *fastidiosa* (strain TemeculaL) on tobacco seedlings, with soil drenching at 500/1000 ppm and 500/500/1000 ppm in 50 mL, reduced bacterial populations by >2 to 3 log_10_ and lowered disease severity by 57–76%. In blueberries infected with *X. fastidiosa* subsp. *Multiplex*, drenching with 1000 ppm (two applications) and 1000/1000/500 in 200 mL, reduced bacterial populations by 1–2 log_10_ and decreased disease severity by 39–43% [153].

#### 5.3.3. Virus

Phyto-fabricated ZnO and SiO_2_ nanoparticles were evaluated against TMV under both in vitro and in vivo conditions. In vitro application of ZnO and SiO_2_ caused breakage and aggregation of TMV within 2 h post-inoculation [154]. Activation of **the** antioxidant defense response was observed in tomato under external application of ZnONPs against tobamoviruses, indicating strong antiviral properties [155]. ZnO and SiO_2_ NP applications induced peroxide production even in the absence of the virus, and both enhanced the activity of antioxidant enzymes such as catalase (CAT) and peroxidase (POD) [156]. A combined effect of plant-based protein 2S albumin and ZnO administered as an albumin protein injection @ 300 ppm and 330 ppm in a 1:1 ratio was tested in an insect-free greenhouse. The formulation showed the best performance against *Candidatus liberibacter asiaticus*, with a significant reduction in bacterial population compared to the initial load at 30, 60, 90, and 120 days after treatment (DAT) [157]. Table 4 represents the antifungal, antibacterial and antiviral affects of different ZnONPs against different microorganisms.

#### 5.3.4. Antimycotic and Mycotoxin Inhibiting Affinity

The antimycotic potential of ZnONPs and their composites was demonstrated against a broad spectrum of plant pathogenic fungi, ranging from lower fungi such as Peronospora and Pythium [164,167] to fungi at perfect and imperfect stages such as *Alternaria*, *Aspergillus* [161,168], *Botrytis cinerea*, *Colletotrichum gloeosporioides*, and vascular wilt pathogens like *Fusarium*, *F. moniliforme*, and *F. oxysporum* [169,170]. At lower concentrations, ZnONPs inhibited fungal spore germination and impacted mycelial growth—e.g., at 3 mM concentration against *B. cinerea* and *P. expansum* [164]. ZnONPs, ZnCl_2,_ and ZnONPs at 15–20 mg L^−1^ concentrations inhibited spore germination of *Peronospora tabacina* [167]. The mode of action of ZnONPs in antimycotic activity is primarily through inhibition of spore germination. Zn particles adsorb onto the fungal hyphae’s surface, causing morphological alterations in the cell wall and membrane, leading to disruption, ion channel leakage, and extensive thinning and branching [170].

## 6. Applications of ZnONPs in Precision Agriculture for Efficient Food Production

Nearly 17% of the global population suffers from Zn deficiency—approximately 1.1 billion people—leading to serious health issues such as poor pregnancy outcomes, stunted growth, weakened immunity, developmental disorders, and delayed recovery from illnesses like COVID-19. FAO and WHO advocate zinc fortification in diets; however, correcting zinc deficiency is challenging. Nearly half of the world’s soils lack sufficient zinc, conventional zinc fertilizers are expensive and inefficient, and developing zinc-rich crops through breeding or genetic methods is complex and time-consuming. Moreover, zinc-rich foods are often unaffordable in low-income regions, where deficiency is most prevalent [171]. Zinc is one of the most important micronutrients for plants, yet Indian soils show widespread deficiency. It is estimated that currently 50% of Indian soils are zinc-deficient, and this is projected to rise to 63% by 2025 [172]. Need-based, site-specific application of nutrients through foliar spraying supports improved Zn concentration in crops through a technology-driven precision agriculture approach [173].

Protected cultivation, an emerging precision agriculture method, addresses food security while achieving target yields with optimized input use. Foliar application of 100 g L^−1^ at the fifth leaf stage significantly reduced plant disease compared to the control in crops like tomato [174] and tobacco [167,175]. Appropriate dosage of ZnO nanoparticles in solanaceous vegetables such as capsicum and tomato is crucial; lower concentrations maximized yield under protected cultivation conditions [176]. Foliar application of nano-zinc on bell pepper leaves under greenhouse conditions showed significant increases in plant height, flower count, and fruit weight—rising 32–29% compared to the control [177].

### Soil Remediation

Soil deterioration caused by extensive pesticide and fertilizer use leads to compaction and weakens soil health. ZnO nanoparticles are being studied for their role in remediating contaminated soils under diverse geological conditions. Immobilization of heavy metals in contaminated soils using ZnO nanoparticles reduces their toxicity, particularly in soils contaminated with cadmium (Cd), lead (Pb), and Zn. The photocatalytic properties of ZnONPs also make them effective in degrading organic pollutants in soil, such as pesticides and polycyclic aromatic hydrocarbons. Studies conducted between 2022 and 2024 have demonstrated the potential of ZnONPs in breaking down these persistent pollutants. Recent research shows that green-synthesized ZnONPs act as efficient adsorbents, significantly improving the elimination of carbamazepine from both soil and water [178]. In addition to remediation, ZnONPs contribute to soil quality by promoting the growth of beneficial microorganisms and enhancing nutrient availability. This dual functionality makes ZnONPs a promising tool for sustainable agriculture and environmental restoration [179,180].

## 7. Safety Measures for Active Delivery of ZnNPs in the Environment

Regulatory authorities issued guidelines on the safety, permissible levels, and handling procedures of ZnO nanoparticles and their utilization. Different international agencies have been established to provide guidance on protective equipment and exposure monitoring [181]. Field applications of ZnONPs and their effects on natural pollinators and honeybees were studied to examine the toxic effects of silver nanoparticle-loaded titanium dioxide (Ag-TiO_2_), titanium dioxide (TiO_2_), and zinc oxide-titanium dioxide (ZnO-TiO_2_) on honeybees (*Apis mellifera*) [182]. Studies indicated that supplementation of nano-Zn at 200 µg L^−1^ in syrup increased the antioxidant status of broods and the body weight of newly emerged bees [183]. Toxicological studies revealed that exposure to ZnO nanoparticles resulted in a decrease in LC_50_ values from 275 to 162.55 mg over 288 h, indicating increasing toxicity over time. Bees fed 500 mg/100 mL experienced the highest mortality, indicating that higher dosages are lethal to honeybees [184]. Neurotoxic studies were conducted to evaluate the effects of ZnONPs and Zn^+2^ ions on honeybee behavioral patterns. Acetylcholinesterase activity increased in bee populations exposed to ZnO NMs and Zn^2+^, indicating neurotoxicity; however, only Zn^2+^ exposure elevated feeding rates. Research findings suggest that zinc ions, whether from salts or nanoparticles, exhibit neurotoxic properties, potentially threatening bee health and overall colony survival [185]. Both beneficial and detrimental effects have been observed in various evaluations of ZnONPs on honeybees. It was found that inclusion of nano-Zn at a dose of 200 µg L^−1^ increased antioxidant status and upregulated the relative gene expression of *vg* and *sod-1*, correlating with incremental dosages of nZn application [183].

**Figure 4:**
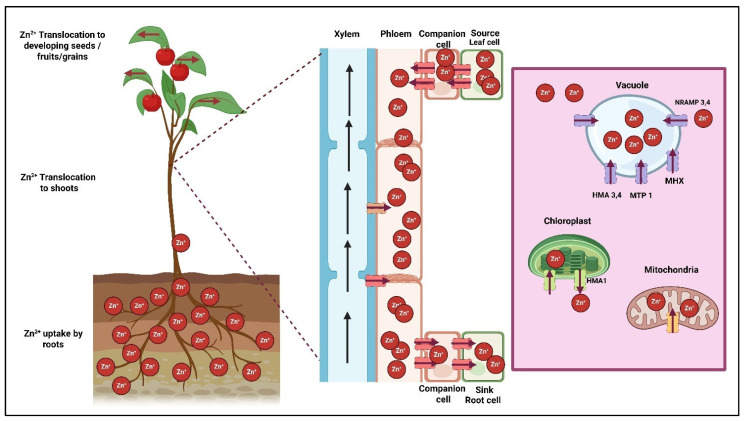
Translocation of nanoengineered ZnONPs and their mobility inside plants is represented in the above image, where different modes of application enhance mobility, with most translocation occurring in phloem cells for transportation from root to aerial parts of the plant, where the translocation of plant-synthesized food storage sinks is located.

To reduce the toxicity effects of ZnO nanoparticles, new synthesis methods such as green synthesis and mycosynthesis are being explored, as they can significantly lower toxicity levels [186,187]. Surface modifications of ZnO nanoparticles, including coating with biocompatible polymers, help limit their reactivity and toxicity [188,189]. Green synthesis of ZnO is considered “safe by design,” with high potential and low environmental risk, as noted by researchers [190]. ZnONPs undergo various transformations such as aggregation, sedimentation, and partial dissolution into Zn^2+^ ions processes influenced by environmental parameters like pH, salinity, and organic matter content. These changes affect their mobility, bioavailability, and toxicity, and their translocation mechanisms under various nanoengineered ZnO forms in plants are illustrated in Figure 4.

## 8. Ecological Impacts

### Toxicity Toward Non-Target Organisms

The effective roles of ZnONPs have been addressed earlier, particularly their ability to help plants overcome abiotic and biotic stress. The small size of the nanoparticles is inherently advantageous for targeted delivery and mode of action. ZnONPs significantly inhibit ammonification, with low DH activity and FDAH observed under different concentrations of ZnONPs; toxicity was higher under acidic conditions, followed by neutral [191]. ZnONPs also influence changes in soil bacterial communities and their diversity at various concentrations compared to TiO_2_ [192]. ZnONPs tend to aggregate in soil, a process influenced by several factors such as soil organic matter (SOM), pH, and soil colloids, all of which affect colloidal stability, key determinants of ZnONPs toxicity in soil [193]. ZnONPs impact plant growth and their symbiotic relationship with VAM; higher concentrations of ZnONPs negatively affect plant growth, along with observed decreases in ROS levels at high dosages (800 mg/kg of soil), resulting in reduced nutrient acquisition and root activity [194]. Toxicity levels of ZnONPs and ionic Zn were evaluated using EC_50_ values, where toxicity in earthworms was identified for ZnONPs in the range of 694–>2200 mg, while for ionic Zn, toxicity was estimated between 277 and 734 mg Zn/Kg [195]. Soil dehydrogenase activity was drastically impaired due to the application of ZnONPs, which lowers the soil respiration rate [196]. NP applications employ various mechanisms that act as antibacterial agents, consistent across all types of bacteria regardless of their biotope. The antibacterial role of ZnONPs is exerted through direct contact with bacterial cells, leading to the disruption of bacterial integrity and the production of ROS, which rapidly accelerates cell destruction [197].

## 9. Future Perspectives and Research Opportunities

### 9.1. Advancements in Ecofriendly Synthesis

Green synthesis of ZnONPs is an important method of NP production; the use of biological systems as reducing agents in green synthesis increases their efficiency with less harm to the environment. Different types of green synthesis mechanisms are being followed by various researchers in the production of green tiny metallic and non-metallic NPs. Mycosynthesis, which is employed in the nanosynthesis of different nanoparticles using various fungal agents, is one of the top methods of the bottom-up strategy, enhancing colloidal structures. These structures are most feasible for obtaining nanoparticles in rods and sheets without errors and with homogenous chemical compositions [198]. Myconanotechnology NP synthesis employs fungal mycelium treated with metallic salt solutions, stimulating fungi to synthesize many enzymes and metabolites for survival. The catalytic effects of these extracellularly produced enzymes reduce the toxicity of metal ions into non-toxic metallic solid NPs. In silver NPs, synthesis through mycosynthesis involves reduced nicotinamide adenine dinucleotide (NADH) and NADH-dependent reductases that reduce Ag^+^ to Ag^0^ [199,200,201].

The botanical extraction method commonly uses biopolymers in the production of ZnONPs. Green synthesis of NPs through botanical extraction is an age-old practice dating back to the 1900s, but the exact procedures and underlying mechanisms are still under investigation [202]. Botanical extracts contain various phytochemical compounds, which act as reducing, stabilizing, and capping agents in the synthesis of different NPs [203]. Green synthesis of ZnONPs using plant extracts follows the methods outlined below. Plant samples are collected, dried to obtain powder, and stored in a refrigerator for further experiments. Later, the powder is dissolved in distilled water and boiled at a specific temperature to obtain extracts, which are further characterized to determine the properties of the nanoparticles. Secondary metabolites present in the purified extracts act as reducing agents in synthesizing nanoparticles from precursor solutions. Green synthesis of ZnONPs through botanical extracts is cost-effective and ecofriendly, reducing the environmental impact of pollutants. Furthermore, toxicity is tested by studying antioxidant and anti-inflammatory activities to understand the effects of these synthesized nanoparticles [204,205].

### 9.2. Future Scope and Research Opportunities

ZnONPs, or Zn metallic nanoparticles, due to their tiny size, enable efficient delivery of the Zn element under various soil pH conditions and improve its bioavailability in plants. The potential antibacterial and antifungal properties of these NPs help reduce pesticide usage for controlling plant diseases and pests. The main drawback in the green synthesis of these ZnONPs is their stability under natural environmental conditions, which raises questions about the effectiveness of the element across different scenarios. Research gaps need to be analyzed to improve the stability of ZnONPs produced through green synthesis. The use of biopolymers capable of producing ZnO nanocomposites, along with other biopolymers, needs to be studied for their efficient application in agriculture. The combined application of ZnONPs with various biocontrol agents has shown remarkable results in enhancing plant growth and development, contributing to improved crop production. Fungal nanotechnology, where fungal metabolites are employed in the synthesis of ZnONPs, has shown promise, but limited information is available on myco-nano-synthesis using various fungi. More exploratory studies are needed to evaluate their efficiency.

## 10. Conclusions

Analysis and review of the literature allowed us to draw the overall conclusion for the efficient utilization of ZnONPs in agriculture across different aspects, as follows:

1. Seed priming: ZnONPs have been studied for their positive effects in enhancing seed germination, showing improved seed vigor and viability. However, the concentration of ZnONPs is crucial in the seed priming process; lower dosages tend to show greater activity, while higher dosages may negatively affect seedling development.
2. The present review highlights the importance of Zn as a master metallic catalyst, playing a key role in many biochemical reactions. It also emphasizes the role of Zn in the production of various phytohormones, which are essential for plant growth and development. The structural importance of Zn is particularly critical in plant resistance mechanisms, as many Zn-domain plant proteins are involved in expressing resistance to both abiotic and biotic stresses.
3. The present review also emphasizes the entry of foliar-applied ZnONPs into the plant system and their response mechanisms in mitigating various abiotic stresses such as drought, salinity, ROS production, and the activation of stress-related enzymes. Their role in overcoming these stresses is clearly outlined.
4. Role and importance of ZnONPs in mitigating biotic stress. The mode of action of these NPs against fungi, bacteria, and viruses has been studied, along with their actinomytic properties and effectiveness in countering myotoxicity, which are now better understood.
5. It is anticipated that this review could further streamline research on innovative methodological alternatives for the delivery of ZnONPs, aiming for efficient application with minimal environmental risks.

## Figures and Tables

**Table 1:** Multifaceted applications of nano-zinc (Zn) in agriculture.

S. No	Application Area	Example/Study	References
1	Fertilizer Efficiency Enhancement	Increased growth and yield in wheat and maize due to improved root development.	[25,26]
2	Pest and Disease Management	Formulation of ZnONPs in sprays for fungal and bacterial infection protection and insect repulsion.	[27]
3	Soil Health Improvement	Improved soil fertility in zinc-deficient soils and better nutrient availability.	[28,29]
4	UV Protection for Crops	Application of ZnONPs on tomato and strawberry plants to reduce UV stress.	[30,31]
5	Seed Germination and Growth Promotion	Faster germination and more robust seedlings in wheat treated with ZnONPs.	[32]
6	Water Management	Development of ZnONPs-based materials for consistent water supply in crops during dry periods.	[33]
7	Nano-Encapsulation of Agrochemicals	Nano-encapsulation of herbicides and insecticides using ZnONPs for enhanced efficacy.	[34]
8	Biotic and Abiotic Stress Tolerance	Application of ZnONPs in soybean for drought resistance and in rice for salinity tolerance.	[35]
9	Nano-biosensors for Precision Agriculture	Development of nano-biosensors using ZnONPs for optimizing agricultural inputs in precision farming.	[36,37]

**Table 2:** Ecofriendly synthesis of zinc nanoparticles from various species and their applications on different crop species for crop improvement.

S. No	Synthesized Plant Species	Crop Species	Changes Observed	Reference
1	*Syzygium aromaticum* L.	*Pisum sativum* L.	Increase the growth and yield as well as productivity of staple crops.	[86]
2	Bio-inspired chitosan and Zn nanoparticles	*Solanum lycopersicum* L.	Suppressed Xoo infections in rice.	[87]
3	Onion Peel Extract	*Vigna radiate* and *Triticum aestivum*	Enhances the seedling growth and germination percentage and fresh as well as dry weight.	[88]
4	*Coriandrum sativum*	*Lycopersicon esculentum*	Show an excellent promotion of enzymatic and metabolic activity to achieve cell elongation.	[89]
5	*Vernonia cinerea*	Tomato seedling	Boost up the growth and development.	[90]
6	*Eucalyptus globules*	Alternaria leaf blotch—in Apple	ZnNPs damage the surface of the fungal hyphae, thereby discharging cellular materials, ensuing in the contraction of hyphae.	[91]
7	*Citrus limetta* Peels Extract	*Solanum tuberosum* L.	Show potential in vitro and in vivo antibacterial activity reduces the fungal infections.	[92]
8	*Cassia renigera* Bark	*Xanthomonas oryzae*	Improvement in germination rate, moisture rate, and growth rate (shoot/root length, number of leaves).	[93]
9	*Citrus limon*	*Dickeya dadantii*	Inhibited growth, swimming motility, biofilm formation, and maceration of sweet potato tubers and likely combat the bacterial pathogen via multiple mechanisms.	[94]
10	*Eucalyptus* *lanceolata* leaf litter	*Zea mays* L.	Increase grain yield compared with the controls.	[95]

**Table 3:** Application of ZnO nanoparticles (ZnONPs) on various crops under abiotic stress with observable changes.

S. No	Nanoparticles	Crop Species	Observed Changes	Reference
1	ZnO (0, 1000, and 3000 ppm)	*Trigonella foenum-graecum*	Reversed salinity-induced consequences	[138]
2	Zn-, B-, Si-, and Zeolite NPs	*Solanum tuberosum* L., *Diamont cultivar*	Increase (Relative Water content) RWC, Proline, Chlorophyll	[139]
3	ZnO (50, 100, and 150 mg/L) and Si (150 and 300 mg/L)	*Mangifera indica* L.	Application of both NPs enhanced leaf NPK content	[140]
4	ZnO (0, 25, 50, and 100 mg/L)	*T. aestivum* L.	Foliar spray enhanced chlorophyll content, and the activities of (Superoxide dismutase) SOD and (Peroxidase) POXs	[141]
5	ZnONPs (50 and 100 ppm)	*Solanum melongena* L.	Improved in uptake of macro- and micronutrients also increased (Relative Water content) RWC in leaves	[142]
6	ZnO (25 mg/L)	*Leucaena leucocephal*	Improved pigments and soluble proteins, reduced peroxidation	[120]
7	ZnO (0, 50, and 100 mg L^−1^)	*G. max*	Improved root and shoot growth	[143]

**Table 4:** Applications of zinc nanoparticles (ZnNPs) on different biotic stresses and their responses.

S. No	Zinc NPs	Pathogen Studied	Impact	Reference
	**Bacterial**			
1	Zinkicide	*X. alfalfae* subsp. *citrumelonis*	7/8-fold lower MIC	[158]
2	ZnONPs	*Xanthomonas axonopodis* pv. *phaseoli*	Reduction in disease severity on pathogen challenge	[151]
3	ZnONPs	*Xanthomonas oryzae* pv. *oryzae*	Antimicrobial agent for bacterial leaf blight of rice	[159]
4	Cu-Zn hybrid NPs	*Xanthomonas perforans (Cu tolerant GEV485)*	Complete inhibition of growth till 24 h of incubation	[160]
	**Fungal**			
5	ZnONPs	*Alternaria alternata*	Mean inhibition rate (EC_50_) range 235 and 848 g/mL higher efficacy compared to ZnSO4	[161]
6	ZnONPs/CS-Zn-CuNPs	*Alternaria alternata*, *B. cinerea*, *R. solani*	Highest mycelial inhibition by chitosan mixed Zn-Cu nanocomposite	[162]
7	ZnONPs	*Aspergillus niger*	Dose-dependent decrease in radial growth diameter	[163]
8	ZnO and CuO NPs	*Pythium ultimum*, *Pythium aphanidermatum*	Inhibition of growth at low concentrations-morphological changes in the hyphae	[164]
9	ZnONPs	*Fusarium graminearum*	Dose-dependent inhibition of fungal growth	[165]
	**Virus**			
10	ZnONPs	TMV	Deactivating TMV and activating plant immunity in Nicotiana benthamiana	[154]
11	ZnONPs	Tomato Bushy Stunt Virus	Activating RNA interference in plants to guard antiviral protection	[166]
